# Manipulating levels of uncertainty in a decision-making task for obsessive compulsive disorder

**DOI:** 10.47626/1516-4446-2023-3432

**Published:** 2024-11-25

**Authors:** Uma Maheswari Ganesh, Lavanya Sharma, Himani Kashyap, Shyam Sundar Arumugham, Janardhan Reddy

**Affiliations:** 1 Department of Clinical Psychology, National Institute of Mental Health and Neurosciences, Bengaluru, India; 2 Department of Psychiatry, National Institute of Mental Health and Neurosciences, Bengaluru, India

**Keywords:** Impulsive behavior, decision making, beads task, uncertainty, obsessive-compulsive disorder

## Abstract

**Objective:**

We investigated whether manipulating levels of uncertainty would influence performance in a decision-making task in people with obsessive-compulsive disorder.

**Methods:**

This case-control study compares Beads Task (measuring reflection/decisional impulsivity) performance and trait impulsivity (Short Urgency-Premeditation-Perseverance-Sensation Seeking-Positive Urgency [UPPS-P] Impulsive Behavior Scale) in patients with obsessive-compulsive disorder (n = 65) and non-clinical controls (n = 45). Differences between groups were assessed with the Mann Whitney *U* test.

**Results:**

The obsessive-compulsive disorder group had significantly fewer draws to decision (U = 1,845, p = 0.019) and less subjective decision-certainty (U = 1,518, p = 0.00) in the low uncertainty (85:15) condition, and higher negative urgency scores (U = 2,163, p ≤ 0.001) and lower sensation-seeking scores (U = 907.5, p = 0.001) in UPPS-P. However, differences in Beads Task performance did not survive correction for multiple comparisons.

**Conclusion:**

Patients with obsessive-compulsive disorder engage in more impulsive decision-making in the lower uncertainty condition than healthy controls, despite low subjective certainty of the decision. They also had higher scores for the trait impulsivity factor of negative urgency. Future studies should explore the contribution of trait impulsivity, as well as symptom severity, anxiety, and decision-certainty, to impulsivity in obsessive-compulsive disorder.

## Introduction

Obsessive-compulsive disorder (OCD) is characterized by repetitive unwanted thoughts and/or behaviors that the individual feels driven to perform. These behaviors and a perceived lack of control is traditionally explained as a tendency towards compulsivity.^1^ However, both impulsivity and compulsivity are evident in OCD patients.^2^ Research demonstrates links between OCD and behavioral addiction models, marked by cognitive impulsivity, risky choices, and skewed probabilistic reasoning.^3^ Individuals with OCD tend to score higher on self-rated measures of impulsivity than controls, particularly regarding cognitive impulsivity, while the findings are inconsistent for motor impulsivity.^3^-^6^ The impulsivity findings contradict the conventional understanding that OCD is harm-avoidant and overcautious behavior.

Although there is some evidence of impulsive decision-making among individuals with OCD on the Beads Task,^3^,^7^,^8^ this may be complicated by the task demands, such as levels of uncertainty, risk, and response conflict. For instance, OCD patients show increased evidence accumulation prior to decision making,^9^ particularly in tasks involving high uncertainty, which is suggestive of a cautious, harm-avoidant decision-making approach. However, heightened error/performance monitoring in OCD patients may contribute to uncertainty and checking even in low-conflict scenarios.^10^ The contribution of impulsivity vs cautiousness to decision-making in OCD may be influenced by the level of task uncertainty, which has received little empirical attention. Therefore, we aimed to assess self-reported impulsivity and evaluate whether individuals with OCD and non-clinical controls had different levels of task uncertainty in decision-making performance (in the Beads Task).

## Methods

### Participants and procedure

A total of 110 participants (65 OCD patients and 45 non-clinical controls) were recruited between September 2021 and August 2022. The patient group was recruited from the OCD Clinic of the National Institute of Mental Health and Neurosciences, India. The OCD sample comprised individuals whose primary diagnosis of OCD was confirmed with the Structured Clinical Interview for DSM-5-Clinician Version,^11^ who were aged 18-50 years and had at least a 7th grade education level. Those with comorbid severe mental illness (schizophrenia or bipolar disorder) or substance dependence (excluding nicotine), schizotypal disorder, or major neurological conditions, such as epilepsy, cerebrovascular diseases, and neurodegenerative conditions, were excluded. Non-clinical controls aged 18-50 years with at least a 7th grade education level were recruited from the community. Those scoring > 6 on the Self Report Questionnaire-20^12^ and those who were diagnosed with major neurological conditions or were first degree relatives of patients with OCD were excluded.

### Measures

#### Beads Task

This task assesses decision-making under uncertainty. The participants completed two versions of the computerized task. The easy/low-uncertainty version consists of two jars with an 85:15 blue to red bead ratio, while the difficult/high-uncertainty version consists of three jars with a 44:28:28 ratio of yellow/orange/pink beads in each jar. Participants are familiarized with the bead distribution probabilities before each task. They were allowed to draw one bead at a time, which would be replaced in the jar after each draw, and must guess which jar the beads are from. The participants could draw up to 30 beads before their guess. However, following a pilot study, in which most participants reached maximum draws, the parameters were modified to introduce a speed/accuracy tradeoff and enhance performance differences across different levels of uncertainty: participants were instructed that the time taken and the number of beads drawn before decision making would be noted. The order of the two versions of the task was counterbalanced among participants. At the end of the task, the participants were asked to rate their decision-certainty on a scale of 1-100. The primary outcome measures were draws to decision (the number of beads viewed before arriving at a decision) and time taken (the total time taken to reach the decision).

#### Short Urgency-Premeditation-Perseverance-Sensation Seeking-Positive Urgency (UPPS-P) Impulsive Behavior Scale^13^

UPPS-P Impulsive Behavior Scale is a measure of impulsivity that covers the dimensions of sensation seeking (tendency to seek out novel and thrilling experiences), lack of premeditation (tendency to not take into account the consequences of actions), lack of perseverance (tendency to have difficulty staying focused on a task that can be long, boring, or difficult), negative urgency (the tendency to act rashly while in an intense negative mood), and positive urgency (the tendency to act rashly while in an intense positive mood). There are four items in each scale, which are rated on a Likert scale from 1 to 4, with higher scores indicating greater impulsivity.

Besides these measures, OCD patients were also assessed for symptom severity using Yale-Brown Obsessive Compulsive Scale^14^ and Clinical Global Impression.

### Statistical analysis

Statistical analyses were conducted using IBM SPSS Statistics 27. The primary outcome variable was the number of beads drawn before a decision and the time taken to complete the Beads Task. The five domains of the UPPS-P Impulsivity Scale were compared between both groups. The Mann Whitney *U* test was used since the outcome variables were not normally distributed (Kolmogorov-Smirnov). Statistical significance was set at p < 0.05.

False discovery rate correction for multiple comparisons was performed for the primary outcome using an online tool (https://www.sdmproject.com/utilities/?show=FDR).

### Ethics statement

This study was initiated after obtaining approval from the institutional ethics committee.

## Results

Table 1 summarizes the demographic and clinical characteristics of the sample. The two groups did not differ significantly regarding age (p = 0.897) or sex distribution (p = 0.670), but they differed significantly in education (p < 0.001) and total Yale-Brown Obsessive Compulsive Scale scores (p < 0.001).

All patients with OCD were taking serotonin reuptake inhibitors. Table 2 shows the comparison between Beads Task performance and trait impulsivity. The OCD group had significantly fewer draws to decision and lower subjective decision-certainty under low-uncertainty conditions, implying that OCD patients are more impulsive and less confident in decision making. However, this comparison did not survive correction for multiple comparisons (p = 0.076). Regarding trait impulsivity, OCD patients had higher negative urgency and lower sensation-seeking than non-clinical controls (p < 0.001) (Table 2). Regression analysis showed no significant clinical/demographic predictors of task performance (Supplementary Table S1).

## Discussion

In this study, we aimed to explore the effect of task uncertainty on decision-making performance in the Beads Task in OCD patients compared to non-clinical controls. Patients with OCD demonstrated lower information gathering on the low-uncertainty version despite reporting lower subjective decisional certainty, which is suggestive of greater impulsivity. In line with previous studies, we found no association between symptom severity and task performance.^3^

These findings contrast with other Beads Task studies, which indicate that patients with OCD require greater information prior to decision-making or no noteworthy difference in task performance compared to controls.^8^ However, in other studies the OCD patients displayed decisional impulsivity in the 85:15 version, which has been linked to overconfidence and reflection impulsivity.^3^-^5^ These inconsistencies denote that sample characteristics and procedural differences might contribute to varied task performance. For example, three out of seven studies had a sample size < 12 and many had OCD patients with minimal comorbidities.^15^ Around 16% of our sample had comorbid mood disorders, which might have influenced our findings. In line with existing findings, there were no significant differences between groups in task performance on the high-uncertainty version of the Beads Task; it has been observed that moderately unambiguous conditions are more sensitive to intolerance to uncertainty than low/highly ambiguous versions of the task.^7^

It is interesting that OCD patients had greater decisional impulsivity only in the low-uncertainty condition, while no differences were observed in the high-uncertainty version. Other authors have also noted that individuals with OCD generally maintain a similar processing approach regardless of whether the situation involves high or low conflict.^16^ The lack of adaptability may result in differing performance in low-conflict situations, while their performance is similar to healthy controls in high-conflict situations.

OCD patients had high self-reported impulsivity in the domain of negative urgency, which has been observed in other studies as well.^17^ It could be hypothesized that negative urgency could be a factor in impulsively arriving at a decision based on less information (beads drawn), despite lower decisional confidence, in order to alleviate the distress, i.e., the anxiety associated with task uncertainty. However, it is unclear under what circumstances this conflict leads to an impulsive vs an overcautious approach to decision making. Impulsivity and compulsivity may exist on opposite ends of a continuum, or as orthogonal constructs. Nevertheless, both constructs are hypothesized to result from failures in top-down cognitive control, which has been consistently observed in OCD patients. While some research indicates that impulsivity and uncertainty tolerance may be trait-like, others suggest they may be modified by treatment,^8^ or even that some impulsivity may be functional/adaptive.

This study has several limitations. We did not control for other executive functions or for affective or motivational states, which may have influenced task performance. All patients were on medications, which could have influenced the results. The task is a single trial condition, which limits comparisons. Although a moderately ambiguous version of the task may have increased uncertainty differences, in a pilot study the 60:40 version did not discriminate significantly between groups. We also did not include a comparison group with other disorders.

Overall, our results suggest that OCD patients have increased negative urgency and decisional impulsivity, which is more apparent in low-uncertainty conditions. Future studies should explore the mediation of trait impulsivity and the role of affective and metacognitive aspects of task uncertainty on decisional impulsivity and the role of impulsivity in OCD treatment.

## Disclosure

The authors report no conflicts of interest.

## Figures and Tables

**Table 1:** Demographic and clinical characteristics of the sample (n=110)

Variables	OCD group	HC group	U/χ*	P-value
Age (years)	29 (9.00)	27 (9.00)	996.50	0.897
Sex	Men = 35, women = 30	Men = 27, women = 18	0.18	0.670
Education (years)	16 (3.00)	18 (3.00)	1479.50	0.00†
Comorbid disorders	Mood disorders (n=11), personality disorders (n= 3), other-generalized anxiety disorder (n=1)	N/A	N/A	N/A
YBOCS total	22 (14.00)	0 (0.00)	112.50	0.00†
CGI	3 (3.00)	N/A	N/A	N/A

Data presented as n/median (interquartile range [IQR]), unless otherwise specified.

CGI = Clinical Global Impression; HC = healthy controls; N/A = not applicable; OCD = obsessive-compulsive disorder; YBOCS = Yale Brown Obsessive Compulsive Scale.

† Chi square for comparing categorical data and Mann Whitney *U* for continuous data.

**Table 2:** Comparison of Beads Task performance and trait impulsivity

Variable/Group (n)	Mean rank	Median (IQR)	U	P-value	FDR corrected value
Draws-to-decision (85:15)					
OCD (65)	49.61	8 (6.00)	1,845	0.019*	0.076
HC (45)	64.01	10 (4.00)			
Draws-to-decision (44:28:28)					
OCD (65)	53.17	21 (14.00)	2,649	0.355	0.474
HC (45)	58.87	23 (9.00)			
Time taken (85:15)					
OCD (65)	51.38	56 (55.00)	1,730.5	0.103	0.206
HC (45)	61.46	32 (20.00)			
Time taken (44:28:28)					
OCD (65)	53.71	64 (53.00)	1,579	0.479	0.479
HC (45)	58.09	72 (42.00)			
Decisional certainty (85:15)					
OCD (65)	34.27	80 (18.00)	1,518	< 0.00*	
HC (45)	56.73	95 (13.00)			
Decisional certainty (44:28:28)					
OCD (65)	37.11	70 (20.00)	1,390	0.002*	
HC (45)	53.89	80 (18.00)			
Lack of premeditation					
OCD (65)	55.83	10 (3.00)	1,441	0.895	
HC (45)	55.02	10 (6.00)			
Lack of perseverance					
OCD (65)	52.55	9 (3.00)	2,689.5	0.240	
HC (45)	59.77	10 (6.00)			
Negative urgency					
OCD (65)	44.72	10 (4.00)	2,163	0.001*	
HC (45)	71.07	13 (6.00)			
Positive urgency					
OCD (65)	52.03	9 (4.00)	2,723	0.167	
HC (45)	60.51	10 (5.00)			
Sensation seeking					
OCD (65)	64.04	11 (6.00)	907.5	0.001*	
HC (45)	43.17	9 (3.00)			

FDR = false discovery rate; HC = healthy controls; IQR = interquartile range; OCD = obsessive-compulsive disorder.

* p < 0.05.
