# Supplementary material for: Manipulating levels of uncertainty in a decision-making task for obsessive compulsive disorder
Source: Braz J Psychiatry. 2024 Nov 25;46:e20233432. doi: 10.47626/1516-4446-2023-3432 (PMC11773319; doi:10.47626/1516-4446-2023-3432)
Supplement: Supplementary file 1 [file bjp-46-e20233432-suppl1.pdf]

http://doi.org/10.47626/1516-4446-2023-3432

Supplementary Table S1 Predictors of draws-to-decision in the 80:20 bead task in the obsessive-compulsive disorder group†

Variable	Beta	P-value
Intercept		0.164
Sex	0.170	0.190
Age	-0.030	0.815
Education	0.232	0.087
YBOCS total	0.222	0.071
Negative urgency	0.137	0.254
Sensation seeking	0.244	0.055

YBOCS = Yale Brown Obsessive Compulsive Scale.
† Multiple linear regression was employed.
